# A minireview on covalent organic frameworks as stationary phases in chromatography

**DOI:** 10.3389/fchem.2024.1384025

**Published:** 2024-03-28

**Authors:** Raquel Gavara, Sergio Royuela, Félix Zamora

**Affiliations:** ^1^ Departamento de Química Inorgánica, Facultad de Ciencias, Universidad Autónoma de Madrid, Madrid, Spain; ^2^ Institute for Advanced Research in Chemical Sciences (IAdChem), Universidad Autónoma de Madrid, Madrid, Spain; ^3^ Condensed Matter Physics Center (IFIMAC), Universidad Autónoma de Madrid, Madrid, Spain

**Keywords:** COF, COF processability, COF composites, COF stationary phase, chromatography

## Abstract

Advances in the design of novel porous materials open new avenues for the development of chromatographic solid stationary phases. Covalent organic frameworks (COFs) are promising candidates in this context due to their remarkable structural versatility and exceptional chemical and textural properties. In this minireview, we summarize the main strategies followed in recent years to apply these materials as stationary phases for chromatographic separations. We also comment on the perspectives of this new research field and potential directions to expand the applicability and implementation of COF stationary phases in analytical systems.

## 1 Introduction

**FIGURE 1:**
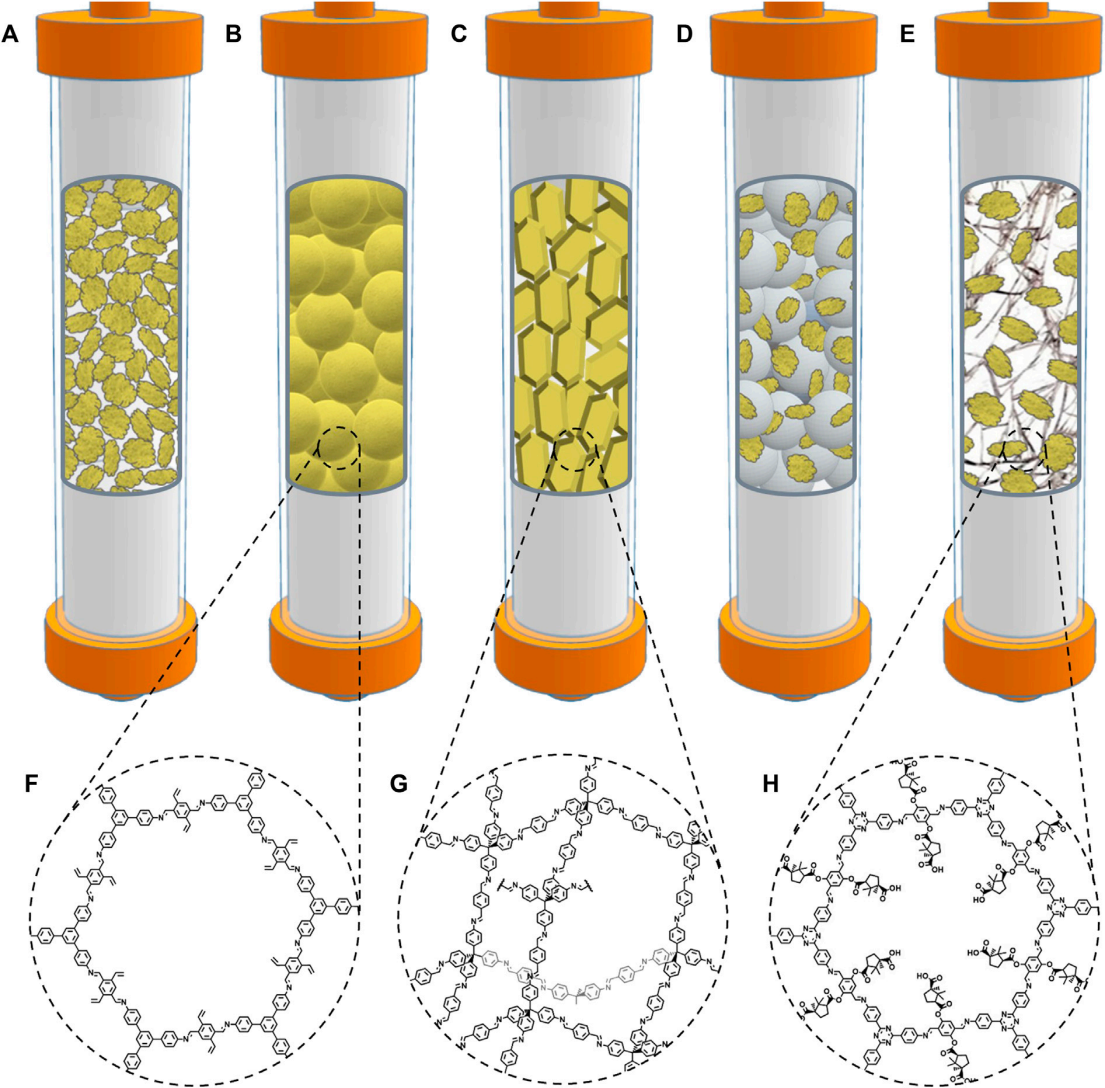
Strategies that have been used to prepare stationary phases based on COF powders: **(A)** polydisperse and irregular COF powders, **(B)** monodisperse micron-sized spherical COF particles, **(C)** single-crystal COF particles, **(D)** physical mixture of irregular and polydisperse COF powder with silica microspheres, and **(E)** irregular COF powder incorporated into porous polymer monolithic columns. COF-V **(F)**, COF-300 **(G)**, and COF CTzDa **(H)** are representative examples of COFs employed in strategies **(B)**, **(C)** and **(E)**, respectively (Zheng Q. et al., 2021; 2022a; Qian H.-L. et al., 2022).

**FIGURE 2:**
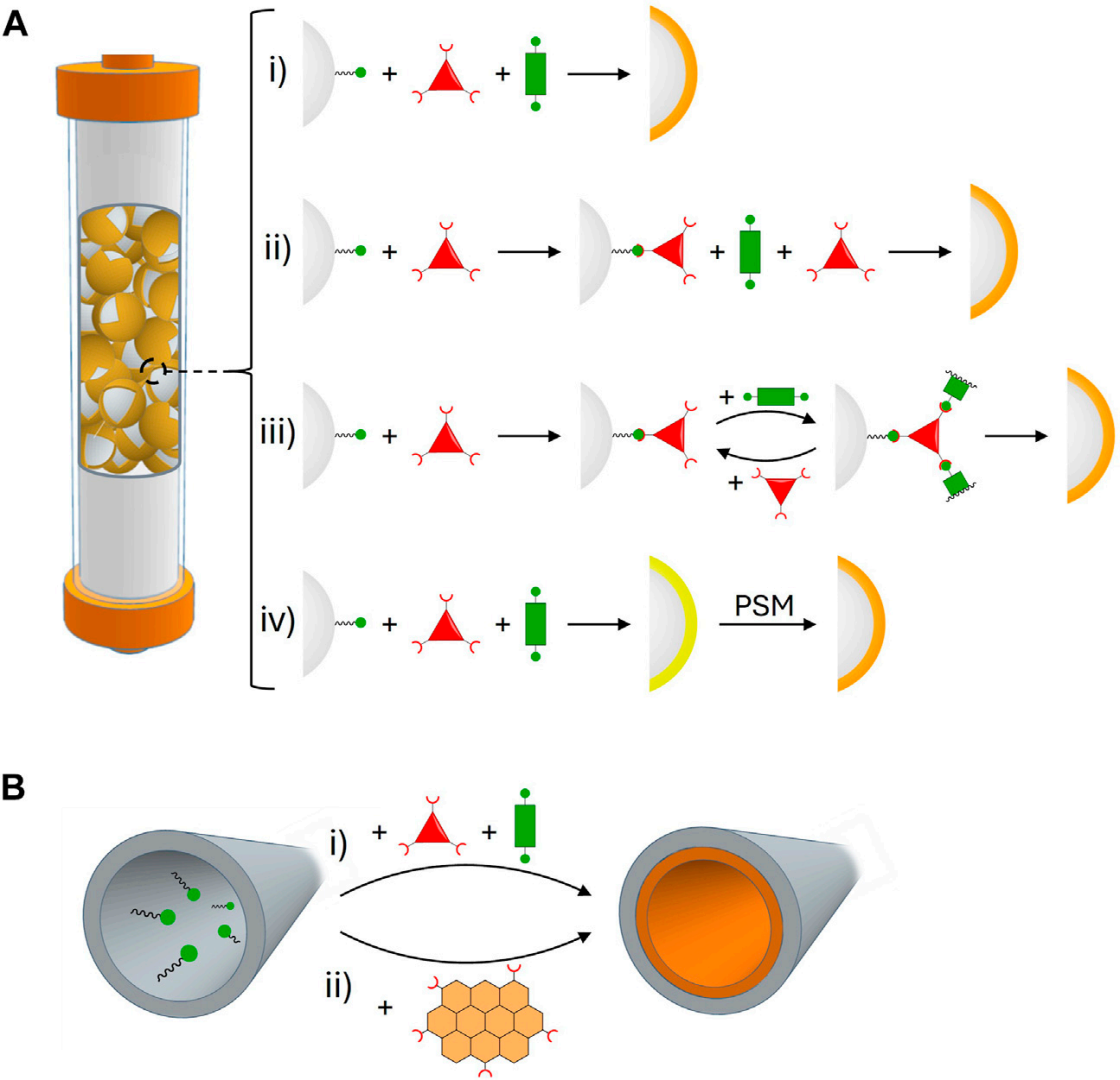
**(A)** Strategies that have been used to prepare stationary phases based on core-shell COF composites with silica microspheres: (i) *in situ* growth, (ii) two-step growth, (iii) layer-by-layer growth, and (iv) *in situ* growth followed by post-synthetic modification. **(B)** Strategies that have been used to prepare stationary phases based on COF chemical coatings: (i) *in situ* growth on the capillary walls and (ii) physical adsorption/covalent attachment of pre-synthesized COF particles.

Covalent organic frameworks (COFs) are highly crystalline organic polymers with large surface area, tunable pore size and geometry, versatile functionalization, and relatively high thermal and chemical stability. Their crystallinity arises from the dynamic nature of the covalent bonds that join the building blocks, which allows the self-healing and error correction of the backbone during the synthesis and gives rise to the most thermodynamically stable structure (Lohse and Bein, 2018; Geng et al., 2020). Due to their outstanding properties, COFs present great potential in several applications such as supercapacitors and batteries (Sun et al., 2023; Tao et al., 2023), gas adsorption (Chen et al., 2022; Li et al., 2023), catalysis (Martínez-Fernández et al., 2022b; Qi et al., 2023), separation (Wang et al., 2020), sensing (Martínez-Fernández et al., 2022a; Yue et al., 2023) and optoelectronics (Keller and Bein, 2021). Moreover, extensive research has been developed on using COFs for membrane-based separations (Burke et al., 2023). Still, the application of these materials in column-based separations has been much less explored. Chromatography is the most widely applied method for accurate analysis and separation in many fields, such as clinical analysis (Kočová Vlčková et al., 2018), pharmaceutical industry (Steinebach et al., 2016), food and beverage testing (Núñez and Lucci, 2020), environmental detection (Yang et al., 2023), and control of industrial chemical processes (Pollo et al., 2018), among others. The most crucial component in a chromatographic system is the stationary phase. In this regard, the separation efficiency depends on establishing several noncovalent interactions between the stationary phase and the analytes. Due to the control on pore size and chemistry, high surface area, and stability, COFs are gaining much attention as stationary phases for chromatographic separations (Zhang et al., 2019; Fikarova et al., 2022). Thus, over the last few years, three main approaches have been developed for the preparation of stationary phases based on COFs: i) direct packing of COF powders in columns, alone or mixed with another material (Figure 1); ii) preparation of composites involving COFs and other materials (Figure 2A); and iii) formation of COF coatings in capillary columns (Figure 2B). This minireview summarizes the recent developments in COF chromatographic stationary phases following these strategies (Table 1). We warn that works that assert the use of COFs as stationary phases without demonstrating their crystallinity and/or porosity have not been included in this revision.

**TABLE 1:** Summary of stationary phases based on COFs.

Strategy	Stationary phase	Crystallinity^a^	BET surface area (m^2^ g^-1^)	Application	Performance^b^	References
COF powder	Lysozyme⊂COF 1	High	103	HPLC separation of racemates of amino acids and chiral drugs	High	Zhang et al. (2018)
	Peptide⊂COF 1	High	-^c^		Low	
	Lysine⊂COF 1	High	-^c^		Failed	
COF powder—spherical particles	SCOFs-2.0 µm	High	364	LC separation of PAHs, anilines, alkylbenzenes, halogenated nitrobenzenes, phthalates, BSA tryptic digest	Failed	Zheng et al. (2021a)
	SCOFs-1.5 µm	High	569		Low	
	SCOFs-1.0 µm	High	856		High/Good	
	SCOFs-0.8 µm	High	1,167		Failed^d^	
COF powder—spherical particles	SF-COFs	Moderate	170	HPLC separation of organic halides, organic compounds with different hydrophobicity and aromatic ring structures	High^e^	Zheng et al. (2022b)
COF powder—single crystals	Single-crystalline	High	602	HPLC separation of positional isomers of disubstituted benzenes, alkylbenzenes, monosubstituted aromatics and PAHs	High	Zheng et al. (2022a)
	COF-300					
Mixture with cellulose derivative	CDMPC@SCOF CSP1	High	108	HPLC separation of racemates and chiral fungicides	Low	Yan et al. (2022)
	CDMPC@SCOF CSP2	High	64		Good	
	CDMPC@SCOF CSP3	High	45		High	
Mixture with SiO_2_ particles	CCOF 5	High^f^	655^f^	HPLC separation of racemic alcohols	Good	Han et al. (2018)
	CCOF 6	High^f^	613^f^		High	
Mixture with SiO_2_ particles	COF 1	High^f^	666^f^	HPLC separation of ethylbenzene and xylene isomers and other benzene derivatives	High	Huang et al. (2019a)
	COF 1-Zn	High^f^	460^f^		Low	
	COF 2	High^f^	701^f^		High	
	COF 2-Zn	High^f^	535^f^		Low	
Mixture with SiO_2_ particles	PFPP-COF-1a	High^f^	874^f^	HPLC separation of PAHs	Low	Wang et al. (2022b)
	PFPP-COF-1b	High^f^	1,027^f^		Low	
	PFPP-COF-2	High^f^	1,698^f^		High^g^	
Mixture with SiO_2_ particles—“Wrapped in Net Method”	CSP-1	High^f^	-	HPLC separation of racemates and chiral drugs	High/Good	Yuan et al. (2022)
	CSP-2	High^f^	-		High/Good	
Mixture with SiO_2_ particles—“Wrapped in Net Method”	CD-PlasmaCOF-CSP-1	High^f^	581^f^	HPLC separation of racemates and chiral drugs	High/Good	Yuan et al. (2023)
	CD-PlasmaCOF-CSP-2	High^f^	335^f^		High/Good	
Mixture with polymer monolith	3D-IL-COF-1	Low	251	HPLC separation of neutral, acidic, basic, and isomers of organic compounds	High^h^	Liu et al. (2021)
Mixture with polymer monolith	CTzDa-monolith	Moderate	252	HPLC separation of racemic amino acids	High^i^	Qian et al. (2022b)
Composites—*In situ* growth	BtaMth@SiO_2_	High	723^f^	HPLC separation of nitroaromatics isomers/β-cypermethrin and metconazole cis-trans isomers	Good	Zhang et al. (2017)
				HPLC separation of β-cypermethrin and metconazole enantiomers	Failed	
Composites—*In situ* growth	CTpBD@SiO_2_	Low	260	HPLC enantiomeric separation of 20 different pairs of enantiomers	Good	Guo et al. (2021b)
Composites—*In situ* growth	β-CD-COF@SiO_2_	Moderate	-	HPLC enantiomeric separation of 24 different pairs of enantiomers	High	Xu et al. (2021a)
Composites—*In situ* growth	SiO_2_@COF	-^j^	306	HPLC separation of alkylbenzenes, PAHs, positional isomers, nucleosides, anilines and sulfanilamides	High	Zheng et al. (2021b)
Composites—Two-step growth	TpBD@SiO_2_	Moderate	385	HPLC separation of PAHs, acidic, basic and aromatic compounds, and nucleobases, nucleosides, and deoxynucleosides	High^h^	Wang et al. (2017)
Composites—Two-step growth	COF-300@SiO_2_	High	254	HPLC separation of PAHs, ethers, aldehydes, ketones, photosensitizers, drugs, alkaloids	High	Chen et al. (2019)
				HPLC separation of substituted benzenes, nucleosides and nucleobases	Good	
Composites—Two-step growth	NPS@TPB-DMTP	High	177	HPLC separation of monosubstituted benzenes, PAHs, alkylbenzenes, anilines and phthalates	High	Xie et al. (2023)
Composites—Layer-by-layer reaction	COF-300@SiO_2_	High	431	HPLC separation of benzene homologues, PAHs and substituted aromatics	High	Qian et al. (2018)
Composites—Post-synthetic modification	Sil-COF	Low	328	HPLC separation of alkylbenzenes and PAHs	Good	Wan et al. (2023)
	Sil-COF-CD	Low	-	HPLC separation of 2-phenylpropionic acid and 1-phenyl-1-propanol enantiomers	High	
Composites—Post-synthetic modification	SiO_2_@rLZU1	Low	194	HPLC separation of PAHs and benzene derivatives	High	Xu et al. (2021b)
				HPLC separation of tar, phenol, ammonia and other substances present in cooking wastewater	Good	
Composites—Post-synthetic modification	COF@CD@SiO_2_	-^j^	298	HPLC separation of enantiomers, positional isomers, alkylbenzenes and PAHs	High	Zheng et al. (2022c)
Dynamic coating	TpBD	Moderate^k^	885^f^	GC separation of alkanes, cyclohexane and benzene, α- and β-pinene, and alcohols	High^l^	Yang et al. (2015)
Chemical coating—*In situ* growth	CTpPa-1	Moderate^m^	146^f^	GC separation of racemates	High^n^	Qian et al. (2016)
	CTpPa-2	Moderate^m^	104^f^		High^n^	
	CTpBD	Moderate^m^	317^f^		High^n^	
Chemical coating—*In situ* growth	BtaMth	Moderate^k^	-	GC separation of alkanes, alcohols, and aromatic positional isomers	High/Good	Huang et al. (2019b)
Chemical coating—*In situ* growth	COF-V	Moderate^m^	-	CEC separation of benzene derivatives, antileptic drugs, herbicides, active ingredients in Chinese medicine	High	Sun et al. (2021)
Chemical coating—*In situ* growth	TAPB-BPTA	Low	-	CEC separation of benzene derivatives, NSAIDs and parabens	High	He et al. (2022)
Chemical coating—*In situ* growth	TpTFMB	Moderate	964^f^	GC separation of isomers of benzene derivatives, alkenes, and acetates	High	Ma et al. (2023)
	TpPa-CF3	Moderate	1,306^f^		High/Good	
Chemical coating—*In situ* growth	SCOF-303 (1.6 µm)	High	-	GC separation of isomers of xylene, dichlorobenzene and pinene	High/Good^o^	Wang et al. (2023)
	SCOF-303 (1.2 µm)	Moderate	-		High^o^	
	SCOF-303 (0.8 µm)	Moderate	-		High^o^	
	SCOF-303 (0.4 µm)	Moderate	-		High^o^	
Chemical coating—covalent attachment	Tf-DHzOH	High^m^	82	CEC separation of amino acids, sulfonamides, tetracyclines, and benzene derivatives	High	Wang et al. (2022a)
Chemical coating—covalent attachment	TFA-TAPB	Low^m^	-	CEC separation of fluoroquinolones	High	Zong et al. (2022)
Chemical coating—covalent attachment	JUC-515	Moderate^m^	-	CEC separation of fluoroquinolones	High	Yin et al. (2023)

^a^ Crystallinity is classified in “High” “Moderate” or “Low” considering the intensity, signal-to-noise ratio, and half-width of the characteristic signals appearing in the XRD diffractograms.

^b^ Performance is classified in “High”, “Good”, “Low” and “Failed” according to the resolution of the peaks in the chromatograms.

^c^ Lower than surface area of Lysozyme⊂COF 1.

^d^ High back-pressure prevented the measurement.

^e^ Better than commercial C18 and pentafluorophenyl columns.

^f^ Pristine COF or as-prepared COF.

^g^ Better than ZORBAX Eclipse PAH column in resolution.

^h^ Better than C18 column.

^i^ Better than Poroshell 120 chiral-T column.

^j^ Data not available.

^k^ Pristine COF heated up to 250 °C.

^l^ Better than HP-5 column.

^m^ Measurement was made on a coating analog.

^n^ Better than β-DEX 225 and Cyclosil B columns.

^o^ Performance increases with the decrease of particle size in the coating.

## 2 Stationary phases based on COF powders

### 2.1 Pure COF powders

The most straightforward approach to using a COF as a stationary phase involves packing the powder material directly on the chromatographic column (Figure 1). In 2018, Ma et al. designed several chiral stationary phases by immobilizing optically active biomolecules into a new imide-linked COF 1 (Zhang et al., 2018). The pristine material was synthesized under solvothermal conditions and exhibited good crystallinity and high Brunauer–Emmett–Teller (BET) surface area. The protein lysozyme, as well as the tripeptide Lys-Val-Phe and the amino acid L-lysine were covalently attached to the material using the -COOH residues accessible on the surface. After functionalization, a remarkable reduction of the surface area was observed. Nevertheless, crystallinity was preserved. The biomolecule-COFs were evaluated as high-performance liquid chromatography (HPLC) stationary phases towards the separation of various racemates. The results showed that Lysozyme-COF exhibited the best chiral separation efficiency for all the tested racemates. Moreover, it was demonstrated that covalent immobilization surpassed the simple adsorption immobilization of the enzyme into the COF.

However, the direct use of COF solvothermal powders is often restricted due to the polydispersion in sizes, irregular shape of COF particles, and their small crystal size. This usually results in high back-pressure and low column efficiency. A way to overcome these issues involves preparing micron-sized COFs with regular spherical shapes. This strategy was employed by Lin and co-workers (Zheng Q. et al., 2021), who prepared micron-sized spherical particles of the imine-linked COF-V (Sun et al., 2017) in a facile synthesis at room temperature. They could synthesize four spherical COFs with tunable sizes of ca. 2, 1.5, 1, and 0.8 μm, by modulating the amount of catalyst used in the synthesis. All the materials showed good monodispersity and high crystallinity. The measured BET surface area was directly related to the amount of catalyst and decreased with the increase in particle size, ranging from ca. 360–1,170 m^2^ g^-1^. After packing the materials in short columns for liquid chromatography (LC) separations, the elution of several aromatic compounds and protein digests revealed an excellent chromatographic performance for the 1 μm particle size COF. It was observed that a higher size led to a decreased resolution and retention. However, a decrease in particle size also led to a noticeable increase in column pressure, which made it impossible to test the 0.8 μm particle size COF. The same research group employed a similar approach to prepare a new HPLC stationary phase based on the column packing of a fluoro-functionalized spherical COF. This COF-packed column exhibited superior performance towards the separation of organic halides than commercial C18 and pentafluorophenyl columns (Zheng et al., 2022b).

Another way of controlling particle size and shape relies on developing single-crystalline COF particles. This approach was recently applied to prepare new HPLC stationary phases (Zheng et al., 2022a). The authors were able to obtain, at room temperature, single-crystalline particles of the 3D imine-linked COF-300 (Ma et al., 2018), which exhibited a regular geometric shape and size of around 10 μm. The material presented an excellent diffraction pattern and high BET surface area. The packed HPLC column showed ideal resolution in separating positional isomers of small aromatic compounds, including nitroaniline, dichlorobenzene, and diethylbenzene. The study also found that the single-crystalline COF-300 exhibited higher resolution and selectivity for positional isomers than commercial columns and a polycrystalline COF-300-packed column.

### 2.2 Mixtures with other materials

The physical mixing of COFs with other materials (Figure 1) can introduce new separation properties in the stationary phases. For example, Cai and co-workers prepared chiral stationary phases by mixing a cellulose derivative with spherical COFs (Yan et al., 2022). Nevertheless, the main goal of physical mixing is usually to overcome the high column back-pressures related to standard COF powders. In that sense, silica is often selected as a co-packing material due to its availability, stability, and easy preparation as monodisperse particles with regular shapes (Qiu et al., 2011). Thus, Cui and co-workers have implemented this approach in several recent investigations (Han et al., 2018; Huang J. et al., 2019; Wang et al., 2022b; Yuan et al., 2022; Yuan et al., 2023).

In 2018, they reported a new 3D chiral COF, CCOF 5, based on the solvothermal reaction of a TADDOL-derived tetraaldehyde with a tetrafunctionalized amine. The post-synthetic oxidation of the imine linkages gave rise to the amide-linked CCOF 6. Both materials presented good crystallinity and high BET surface area. The HPLC-packed columns were prepared by mixing ∼0.3 μm COF particles with silica microspheres with an average size of 5 μm. Several alcohol racemates were selected to evaluate the chromatographic properties of the columns. The experiments revealed superior resolution performance for the amide-linked COF compared to the pristine material, which was attributed to stronger interactions between the amide groups and alcohol guests in the pore channels (Han et al., 2018).

COFs have also been incorporated into porous polymer monolithic columns to reduce column back-pressure (Svec and Lv, 2015; Song et al., 2023). This strategy was recently employed by Yan and co-workers, who prepared the 3D imine-linked 3D-IL-COF-1 via solvothermal process instead of the previously reported ionothermal synthesis (Guan et al., 2018). The resulting material exhibited a notably high BET surface area and good crystallinity. The COF powder was mixed with the methacrylic precursors of the monolithic polymer and introduced in the HPLC column, where the polymerization reaction was carried out. The characterization of the COF-monolith showed an evident decrease in the BET surface area, which nevertheless was higher than the corresponding blank monolith, pointing out the positive contribution of the COF to the porosity. The efficiency of the HPLC column was tested towards the separation of neutral, acidic, and basic small organic compounds, as well as isomers of disubstituted benzene derivatives and polycyclic aromatic hydrocarbons (PAHs). The chromatographic performance was, in all cases, superior to that found for the blank monolith. Moreover, a comparison with a commercial C18 column revealed that the COF-monolith column was better for separating disubstituted benzene and PAH isomers (Liu et al., 2021). Qian and co-workers developed a HPLC stationary phase using a similar approach based on encapsulating the chiral imine-linked COF CTzDa (Liu et al., 2020) into a methacrylic monolithic polymer. The column gave better enantioseparation of several amino acids than a commercial chiral column (Qian H.-L. et al., 2022).

As the main conclusions of this section, we can say, on the one hand, that the use of pure COF powders with controlled geometry and size alleviates the problems associated with high column back-pressures, although there are still issues when the particle size is small (sub-micrometric range). This could be inconvenient when dealing with samples of difficult separation since decreasing the particle size is usually related to a better resolution performance. On the other hand, using mixtures with other materials, namely silica particles and polymer monoliths, has also been demonstrated to be beneficial in decreasing column back-pressure. However, this improvement is achieved at the expense of losing part or most of the pivotal properties of COFs, i.e., crystallinity and surface area. New research efforts should focus on attaining suitable column back-pressures with minimum loss of these essential attributes of COFs.

## 3 Stationary phases based on COF composites

As mentioned in the previous section, COF powders as stationary phases for chromatography usually present the drawbacks of their wide particle size distribution and the irregularity of their shape, leading to inefficient packing, high column back-pressure, and poor separation performance. Packing the columns with COF–silica mixtures helps reduce these issues and it uses less active material, but can suffer from COF leaching during column runs. To avoid this problem, covalent COF@SiO_2_ core-shell composites can be prepared to immobilize COFs on the surface of silica microspheres (Figure 2A). This methodology has been successfully accomplished via four different strategies.

### 3.1 *In situ* growth method

The most straightforward approach to preparing these core-shell composites consists of adding silica microspheres functionalized with amine groups on their surface (SiO_2_-NH_2_) to the COF synthetic medium. Thus, as the COF forms, it is simultaneously anchored to the surface of the silica microspheres via the same covalent bonds that act as COF linkages. This has been achieved with hydrazone-based (Zhang et al., 2017), imine-based (Xu N. Y. et al., 2021; Guo P. et al., 2021), and imide-based COFs (Zheng Y. et al., 2021).

In a first work, Zhang and co-workers used this approach to immobilize the novel hydrazone-based BtaMth COF on the surface of 5 μm SiO_2_-NH_2_ microspheres (Zhang et al., 2017). The COF is a variation of COF-42 (Uribe-Romo et al., 2011) endowed with chiral side chains. The microspheres were added directly to the solvothermal reaction mixture, resulting in a core-shell composite that maintained high crystallinity and high porosity. The BtaMth@SiO_2_ composite was used to pack HPLC columns, which showed high-resolution performances for separating the positional isomers of nitrotoluene and nitrochlorobenzene in the reverse-phase mode. Moreover, the separation of the *cis-trans* isomers of β-cypermethrin and metconazole in the normal-phase mode could be achieved. However, the separations of the enantiomers of these two analytes on the BtaMth@SiO_2_ packed column failed.

This strategy has also been applied to chiral imine-based COFs by Xie and collaborators (Guo P. et al., 2021). They prepared a novel chiral monomer by esterification of 1,3,5-triformylphloroglucinol (Tp) with (+)-diacetyl-L-tartaric anhydride, and this monomer was then reacted with benzidine (BD) in the presence of 5 μm SiO_2_-NH_2_ microspheres under reflux conditions. The resulting material presents a COF coating of around 250 nm, as determined by scanning electron microscopy (SEM), while slightly improving the BET surface area of the pristine microspheres. The material was used as chiral stationary phase for the HPLC enantioseparation of 20 different pairs of enantiomers, bearing different functional groups, with high enantioselectivity and good reproducibility. Furthermore, the material proved useful for determining enantiomeric excess in commercial reagent samples, obtaining results that agreed with their labeled enantiopurities.

### 3.2 Two-step growth method

A slightly more sophisticated version of the *in situ* growth approach involves a first reaction of the SiO_2_-NH_2_ microspheres with the aldehyde monomer of the COF (Wang et al., 2017; Chen et al., 2019; Xie et al., 2023). This way, complete coverage is achieved in the first layer of the shell, reducing the formation of irregular and self-aggregated COF on the surface of the silica microspheres.

The first example of this approach was reported by Yan and co-workers (Wang et al., 2017). They reacted 5 μm SiO_2_-NH_2_ microspheres with the aldehyde Tp, and the so-formed SiO_2_-CHO microspheres were introduced in the reaction under reflux between Tp and the amine BD that gave rise to the COF TpBD (Biswal et al., 2013), to finally obtain TpBD@SiO_2_ composites with COF shell thicknesses between 50 and 150 nm. These materials retained the crystallinity of the COF, and the BET surface area was significantly improved compared to the pristine microspheres. The TpBD@SiO_2_ composites were packed as stationary phases for HPLC separation of a variety of analytes, including neutral (toluene from ethylbenzene, PAHs), acidic (hydroquinone, *p*-cresol, and *p*-chlorophenol), and basic (nucleobases, nucleosides, and deoxynucleosides) molecules. They showed good column efficiency, high resolution, and good reproducibility, and in the case of the basic molecules, they outperformed the standard C18 column in separation resolution.

### 3.3 Layer-by-layer reaction

To ascertain complete control over the coating thickness, Yan and collaborators applied a layer-by-layer approach to preparing COF@SiO_2_ composites (Qian et al., 2018). With this strategy, the SiO_2_-NH_2_ microspheres first react with the aldehyde monomer, then the amine monomer is added, then the aldehyde again, and so on. This sequential addition of the monomers to the reaction medium ensures complete surface coverage in each step, and it allows fine control over the imine-COF coating thickness by changing the number of monomer addition cycles. The authors employed this methodology to prepare COF-300@SiO_2_ core-shell composites with high crystallinity and BET surface area, which were successful in the HPLC separation of mixtures of benzene homologs, such as benzyl alcohol, aniline, phenol, and methyl benzoate; substituted aromatics, including nitrophenol, nitroaniline, and aminophenol; and PAHs.

### 3.4 Post-synthetic modification

Post-synthetic modification (PSM) is a potent approach to introduce functionality into COFs after they have been formed, thus avoiding incompatibilities between the desired functionalities and, for instance, the reaction medium used to obtain the COF (Segura et al., 2019).

This approach has been used by different research groups (Zheng Y. et al., 2022; Wan et al., 2023) to introduce chiral β-cyclodextrin (β-CD) moieties. Thus, Wang and co-workers prepared silica microspheres modified with epoxide groups (Wan et al., 2023), which were used to anchor the imine-linked COF-V (Sun et al., 2017) onto the surface of the microspheres in a one-pot reaction with the COF monomers. Then, they performed a PSM via thiol-ene click chemistry to attach a thiolated β-CD to the vinyl moieties of the COF. The so-obtained chiral material presented low crystallinity and moderate surface area. However, when packed into an HPLC column, it achieved baseline enantiomeric separation of two chiral analytes, 2-phenylpropionic acid and 1-phenyl-1-propanol. In contrast, the composite before the PSM treatment or a commercial β-CD column failed to separate these compounds.

Finally, Li and co-workers reported a different use of the PSM approach; instead of attaching new moieties to the framework, the authors made a post-synthetic imine-to-amine linkage reduction (Xu S. et al., 2021). Hence, they first covered SiO_2_-NH_2_ microspheres with the imine-based COF LZU1 (Ding et al., 2011) and then reacted them with NaBH_4_ to realize the imine-to-amine linkage transformation. Although the final material exhibited low crystallinity and porosity, it had enhanced stability towards hydrolysis during application as a stationary phase. It showed high performance and good reproducibility in the separation of acidic (phenol, pyrocatechol, and pyrogallol), basic (aniline, 4-chloroaniline, and 4-nitroaniline), and neutral (benzene homologs) compounds, and it was also applied in the separation of real samples, such as tar, phenol, ammonia and other substances present in coking wastewater.

In conclusion, the development of covalent COF@SiO_2_ core-shell composites offers a promising approach to overcome the issues associated with COF powders. While straightforward and one-pot, the *in-situ* growth method lacks precise control over the COF coating. In contrast, growing the COFs in steps or employing a PSM affords a finer control of the material and allows tuning of the properties, at the expense of being more complex to implement. Ongoing research aims at a straightforward methodology to control the thickness and functionalization of the COF shell, which would facilitate achieving baseline separation of racemates and complex mixtures.

## 4 Stationary phases based on COF coatings

Several COFs have also been coated on capillary columns to produce stationary phases for gas chromatography and electrochromatography (CEC) separations (Mao and Chen, 2019; Mametov et al., 2021). The coatings are usually achieved by employing thermal treatments at high temperatures and/or using chemical reactions to anchor the COF to the column surface (Figure 2B). These treatments can affect the crystallinity and porosity of the coated materials; however, in this minireview, we focus on the works where direct or indirect analyses support the preservation of the COF crystalline structure in the coating. For further information on non-crystalline coatings or works in which crystallinity is not reported, we direct the readers to some selected references (Wang et al., 2019; Wang et al., 2022c; Guo J.-X. et al., 2021; Natraj et al., 2022; Yuan et al., 2022; Yuan et al., 2023; Yusuf et al., 2023).

### 4.1 Dynamic coating

In 2015, Yan and co-workers reported the preparation of the imine-linked COF TpBD as spherical micron-sized particles using a facile synthesis at room temperature (Yang et al., 2015). The material showed moderate crystallinity, although the authors confirmed that it was preserved up to 250°C. Moreover, the as-synthesized COF presented a high BET surface area. The COF coating on the capillary column was achieved by the dynamic coating method, using a final thermal treatment at 150°C, which was well below the limit temperature to preserve the crystallinity of the COF. The dynamic coating is primarily based on the physical adsorption of the stationary phase on the inner walls of the capillary column (Horvath and Dolník, 2001). Nevertheless, given the high temperature used in the final step, the occurrence of some type of covalent attachment of the COF on the column surface cannot be ruled out. The coated column showed good separation properties towards small organic molecules, such as alkanes, cycloalkanes, benzene, and alcohols. Moreover, it exhibited better performance than a commercial HP-5 column.

### 4.2 Chemical coating

Chemical coating implies the COF’s covalent immobilization to the capillary column’s inner surface. This approach can render more stable stationary phases than a dynamic coating, which occasionally may require regeneration of the column (Horvath and Dolník, 2001). To date, two main strategies have been applied to prepare chemical coatings of COFs on capillary columns. The first one is based on the *in situ* growth of the COF on the column walls (Qian et al., 2016; Qian et al., 2022 H. L.; Huang X. L. et al., 2019; Sun et al., 2021; He et al., 2022; Ma et al., 2023; Wang et al., 2023). This methodology was utilized by Yan and co-workers in 2016 to develop chiral COF-bound capillary columns for chiral gas chromatography. The authors first prepared three chiral imine-linked COFs, namely CTpPa-1, CTpPa-2, and CTpBD, as powders outside the columns by reacting a chiral derivative of the aldehyde Tp with several aromatic linear diamines. Characterization of the materials revealed moderate crystallinity and low to moderate BET surface areas. The COF-bound capillary columns were then fabricated by reacting the monomer mixtures in columns modified with 3-aminopropyltriethoxysilane (APTES), to promote the attachment of the aldehyde and subsequent growth of the COF on the column walls. The same type of coating was made in a fused silica plate to confirm the COF structural preservation. The X-ray powder diffraction analysis of the material revealed that crystallinity was maintained. The columns exhibited good chiral separation properties for racemates of small organic compounds, with superior performance to several commercial chiral capillary columns (Qian et al., 2016).

The second strategy to obtain coatings of COFs chemically bound to capillary columns is based on the covalent attachment of the pre-synthesized COF particles (Wang F. et al., 2022; Zong et al., 2022; Yin et al., 2023). Following this approach, Chen and co-workers prepared a stationary phase for open tubular capillary electrochromatography (Wang F. et al., 2022). The stationary phase was based on the hydrazine-linked COF Tf-DHzOH obtained through the reaction of an aromatic linear dihydrazide and 1,3,5-triformylbenzene under solvothermal conditions. The analysis of the as-synthesized material revealed an excellent crystallinity and low BET surface area. Coating of the COF on the capillary column was achieved by reacting a suspension of the COF particles containing residual hydrazide groups with 3-glycidoxypropyltrimethoxysilane (GLYMO) units attached to the inner surface of the column. Analysis of an analogous coating prepared on a glass surface corroborated that crystallinity is preserved in the coating. The column separation properties were evaluated using as analytes amino acids, sulfonamides, tetracyclines and benzene derivatives, and the results showed a better performance of the COF column as compared to the bare capillary and other reported coated capillary columns.

Therefore, coating COFs on capillary columns opens new opportunities for gas and electrochromatography applications. However, it is still challenging to determine if the crystallinity and surface area of the pristine COFs is preserved in the prepared coatings and under the working conditions of gas chromatography. New research should also focus on optimizing the control of coating thickness and roughness.

## 5 Conclusion and future outlook

There are various types of porous materials that, due to their properties, can be attractive alternatives to classical stationary phases based on silica and organic polymers. In particular, metal-organic frameworks (MOFs), COFs, and zeolites present excellent designability, which offers a fine control on chemical functionalization and pore size distributions, which are found typically in the micropore and mesopore range (Slater and Cooper, 2015). The control at the molecular level cannot be achieved with other conventional materials, and their application as stationary phases can significantly improve the separation efficiencies for numerous substrates. Comparing MOFs, nitrogen-based COFs, and zeolites, the former present lower chemical stability, while zeolites are challenging to process. In contrast, nitrogen-based COFs are characterized by high chemical and thermal stabilities. Moreover, several methodologies reported in the last years have facilitated their processing for real-world applications (Martín-Illán et al., 2023). Altogether, these properties make COFs excellent candidates for the development of chromatographic stationary phases, and they can bring solutions in enantiomeric separation of bioactive molecules for pharmaceutical applications, extraction and purification of natural products, and fine chemistry.

Current strategies to prepare COF stationary phases, mainly based on the column packing with controlled shape and size particles, mixtures, and composites, and COF-coating on capillary columns, have demonstrated good to excellent results in the chromatographic separation of several substrates, including basic, neutral and acidic small organic molecules, positional isomers, racemates, and PAHs. Therefore, it is worth continuing to investigate these approaches. However, each of these strategies has some drawbacks, such as the usual increase in back-pressure when sub-micron-sized particles are employed, the loss of surface area of the pristine material in mixtures and composites, and the complex and long synthetical procedures associated with the preparation of some composites and coatings. In this regard, work on new processing methodologies can help to overcome some of these disadvantages. Moreover, conducting more research on the separation and purification of added-value compounds would be interesting, which could open new business opportunities and academia-industry collaborations.
